# The Double Edge Sword of Testosterone’s Role in the COVID-19 Pandemic

**DOI:** 10.3389/fendo.2021.607179

**Published:** 2021-03-16

**Authors:** Johnny S. Younis, Karl Skorecki, Zaid Abassi

**Affiliations:** ^1^ Reproductive Medicine, Department of Obstetrics and Gynecology, Baruch Padeh Medical Center, Poriya, Israel; ^2^ Azrieili Faculty of Medicine in Galilee, Bar-Ilan University, Safed, Israel; ^3^ Department of Physiology, Rappaport Faculty of Medicine, Technion, Haifa, Israel; ^4^ Laboratory Medicine, Rambam Health Care Campus, Haifa, Israel

**Keywords:** COVID-19, age-related, hypogonadism, SARS-CoV-2, testosterone, ACE2, MPRSS2

## Abstract

COVID-19 is a complex disease with a multifaceted set of disturbances involving several mechanisms of health and disease in the human body. Sex hormones, estrogen, and testosterone, seem to play a major role in its pathogenesis, development, spread, severity, and mortalities. Examination of factors such as age, gender, ethnic background, genetic prevalence, and existing co-morbidities, may disclose the mechanisms underlying SARS-CoV-2 infection, morbidity, and mortality, paving the way for COVID-19 amelioration and substantial flattening of the infection curve. In this mini-review, we focus on the role of testosterone through a discussion of the intricate mechanisms of disease development and deterioration. Accumulated evidence suggests that there are links between high level (normal male level) as well as low level (age-related hypogonadism) testosterone in disease progression and expansion, supporting its role as a double-edged sword. Unresolved questions point to the essential need for further targeted studies to substantiate these contrasting mechanisms.

## Introduction

Almost one year has elapsed since the outbreak of the coronavirus disease 2019 (COVID-19) pandemic, caused by the novel severe acute respiratory syndrome coronavirus (SARS-CoV-2). The cumulative number of infected cases and death toll around the world continues to rise. As of January 1, 2021, the number of confirmed global cases of SARS-CoV-2 is 81,658,440 and the number of established human deaths is 1,802,206 cases, reported to WHO, while the numbers continue to evolve. The availability of effective vaccines brings hope for an end to the pandemic, though limitations of distribution might require a year or more to achieve global control. Accumulating evidence suggests that male infection is predominant (1, 2), especially in cases above 60 years of age and specifically in critically ill adults (2–5). Furthermore, intensive care unit admissions and mortality rates are far higher for male than female patients, independent of age (2–5).

Accumulating data from around the globe also shows that the incidence of COVID-19 is strikingly variable in different populations and various ethnic backgrounds, with diverse heterogeneity in virulence (6, 7). Evidence emerging from the United States and England shows that COVID19 mortality is disproportionately high amongst African Americans, Black, Asian, and other ethnic minority communities. Estimates suggest that American counties where Black residents are in the majority have almost six times the rate of death due to COVID19 compared to counties with predominantly white residents (7–9). Socioeconomic and lifestyle factors seem also to be implicated in COVID-19 severity and gender differences (10–13). The likelihood of SARS-CoV-2 infection is significantly higher among minority ethnic communities even after adjustment for important socio-demographic and co-morbidity factors (14).

Furthermore, the incidence of COVID-19 before puberty is particularly low, and even when presenting it is generally mild (1, 15). Contrary to adults, there is no significant gender difference in young patients (16).

Taken together, epidemiological data continue to accumulate since the outbreak of the novel COVID-19 pandemic that suggest the possibility that sex hormone differences between males and females, specifically testosterone levels, normal male and age-related hypogonadism, in addition to genetic factors between different ethnic communities, may play a crucial role in the occurrence, pathogenesis, severity, and subsequent mortality of COVID-19.

## SARS-CoV-2 Pathogenesis of Infection

The entire cell cycle of SARS-CoV-2 infection has been lately elucidated and clarified at the molecular level (17). It is currently well accepted that the port of entry of SARS-CoV-2 to the lungs, as to other vital organs of the body, is *via* the angiotensin-converting enzyme 2 (ACE2) receptor. This receptor is a key element of the renin-angiotensin-aldosterone system (RAAS), a cardinal endocrine/metabolic axis that regulates blood pressure and fluid balance. ACE2 is responsible for the generation of angiotensin 1-7 from angiotensin II. The angiotensin 1-7-Mas receptor axis provokes beneficial balancing and salutary actions to counterpart the adverse actions of the ACE/angiotensin II/AT1R pathway in vital organs, such as the lung, heart, and kidney (18, 19). Therefore, being a receptor for SARS-CoV-2 penetration of host cells, ACE2 integrity plays a crucial protective role against lung and other vital organ injuries (20). Coronavirus mediated ACE2 receptor down-regulation may escalate the counter-part impact of the renin-angiotensin I-angiotensin II-AT1R axis and contribute to the deleterious hyper-inflammatory response of COVID-19 in the lungs (21, 22). Yet, it has been shown that this effect does not hold in all parts of the body (23, 24).

SARS-CoV-2 is enveloped with a single-stranded positive sense RNA genome. The viral envelope bears transmembrane spike proteins (S) as well as other proteins (25). SARS-CoV-2 and SARS-CoV employ the same receptor-binding domain, *via* their surface S glycoproteins, to attach to the ACE2 receptor (26, 27). The S proteins of both viruses have been shown to have a high rate of homology, possess almost identical 3-D structures, and share 76.5% identity in amino acid sequences (28), although the affinity of SARS-CoV-2 to ACE2 has been revealed to be 10 to 20- fold higher than that of SARS-CoV (29). The S proteins have two fused binding subunits, S1 and S2 respectively. The first, S1, is responsible for the virus surface attachment to the host cell, and the second, S2, for the fusion of viral and cellular membranes and viral internalization into the host cell (17). Viral infection and entry to host cells require S protein priming by cellular proteases, which entails S protein cleavage at the S1/S2 and the S2 sites (17). Spike protein priming and cleavage are triggered by the host cellular transmembrane protease serine 2 (TMPRSS2) (17, 30–32). SARS-CoV-2 host cell entry was shown to be blocked by the clinically validated inhibitor of TMPRSS2 - Camostate (17). The priming process by TMPRSS2 seems to be vital for the entry of SARS-CoV-2 into human host cells, and thus plays an integral role in COVID-19 infection and disease progression. Moreover, TMPRSS2 may also cleave ACE2 thus augmenting viral entry (33).

More recently, it has been demonstrated that the S protein of SARS-CoV-2 infects lung host cells by a two-step activation mechanism. A pre-cleavage of the S proteins at the S1/S2 site by furin proteases is essential for subsequent S protein priming and activation (at the S2 site) by TMPRSS2 lung cells (33, 34). This mechanism explains the fusion of infected cells with non-infected cells, which might allow the virus to spread in the body without leaving the host cell. Furin, encoded in humans by the *FURIN* gene, is an enzyme that belongs to the subtilisin-like proprotein convertase family. The latter consists of a family of nine serine secretory proteases that regulate various biological processes in both healthy and disease states (35, 36). Furin is a calcium-dependent serine endoprotease that can efficiently cleave precursor proteins at their paired basic amino acid processing sites.

## High Testosterone Impact on COVID-19 Severity—The TMPRSS2 Connection

TMPRSS2 is a cell-surface protein expressed by the epithelial cells of specific tissues including those in the aero-digestive tract. It is a member of the type II Transmembrane Serine Proteases (TTSPs) family that are involved in multiple physiological and pathological processes, including viral infections and cancer, although its exact physiological role is still under investigation. *TMPRSS2* transcription is regulated by the androgen receptor (AR) (37). Specifically, AR activity is considered a requirement for the transcription of the *TMPRSS2* gene because no other known *TMPRSS2* gene promoter has been described in humans to date (37).

The human AR, located on the X chromosome, functions as a steroid hormone–activated transcription factor, which signals through classical and non-classical signaling pathways (ligand-dependent and independent actions) (38, 39). Androgens can work along three known paths, by intracellular conversion of serum testosterone into dihydrotestosterone (DHT), by testosterone itself, or by intracellular conversion of testosterone to estradiol through aromatization. The AR has widespread expression in many cells and tissues with a diverse range of biological actions involving the development and maintenance of the reproductive, musculoskeletal, cardiovascular, immune, neural, and haemopoietic systems (39, 40).

Androgen receptor expression is low prior to pubertal maturation and may contribute to the low incidence of severe COVID-19 infection in children (41, 42). In addition, the lower rate of severe COVID-19 infection in female patients may be attributed to lower AR expression (43, 44). AR contains two polymorphic nucleotide repeats, GGN and CAG, encoding for glycine and glutamine, respectively (45). Several mutations or polymorphisms have been described in the gene encoding the AR in a variety of diseases or among various ethnic groups. Some of these mutations/polymorphisms are associated with functional changes in the AR expression and mutations in or around the receptor (46–48). Testosterone’s biological action is dependent on the length of the CAG repeat of the androgen receptor gene (49).

Androgen mediated expression of ACE2 and TMPRSS2 may explain the gender difference in COVID-19 disease severity and mortality (50). Furthermore, the frequency of genetic variations in the AR differs by ethnicity, which may suggest a possible explanation for the wide differences in COVID-19 severity and mortality rates between countries and between different ethnic backgrounds in the same country (51, 52).

Various experimental data in mammalian animal models, as well as in numerous, unrelated clinical manifestations, in diverse in-vivo as well as human clinical settings, support the interplay between SARS-CoV-2 and sex hormones, specifically testosterone and AR, most likely *via* the cell host TMPRSSE2.

In animal models, ACE and ACE2 activity in cardiac cells were significantly higher in male compared to female rats, whereas orchiectomy decreased the activity of these enzymes and ovariectomy increased ACE2 but did not change ACE activity (53). In addition, androgen administration to a lung adenocarcinoma cell line up regulated the TMPRSS2 transcript more than two-fold, accompanied by an androgen dependent loading of the AR protein onto the TMPRSS2 enhancer (54). Furthermore, TMPRSS2 inhibition or knock down has been shown to reduce SARS-CoV infection *in vitro* (33).

Just recently, in-vitro studies employing human embryonic stem cell-derived cardiac cells and lung organoids have substantiated that testosterone regulates SARS-CoV-2 development, intensifying its severity in men. Furthermore, the pharmacological dampening of testosterone activity by inhibitors of 5 alpha reductases can reduce ACE2 levels in the target cells, leading to the decay of SARS CoV-2 infectivity (55).

In the clinical setting, recent preliminary studies suggest a high incidence of androgenic (androgenetic) alopecia among male and female patients hospitalized due to severe COVID-19 (56, 57). Androgenic alopecia, often referred to as male pattern (scalp) hair loss, is the most common form of hair loss among men and is associated with AR polymorphism (58). In one small-scale study, clinically significant androgenic alopecia was shown to complicate 71% of males with COVID-19 as compared to 31-57% in literature controls (57).

The androgen-dependency of TMPRSS2 activity is normally expressed at its highest level in the prostate epithelium, as evidenced by several fold abundance compared to all other body tissues (59, 60). While the physiological role of TMPRSS2 is still under investigation, it is significantly up regulated in men with a prostatic disease, including those with prostate cancers (37). Elevated free testosterone was recently shown in a large scale study to be associated with COVID-19 complications in this subgroup of men (55).

Men with metastatic prostatic cancer are usually treated with androgen-deprivation therapies to control the disease. It is noteworthy that these therapies substantially decrease the levels of TMPRSS2. While cancer patients have an increased risk of SARS-CoV-2 compared to non-cancer patients, it has been recently shown by two independent preliminary studies that prostate cancer patients receiving androgen-deprivation therapies are partially protected from SARS-CoV-2 infections (61, 62), supporting further the deleterious role of androgens in the pathogenesis of COVID-19. The active controversy surrounding this topic in contemporary literature calls for well-designed targeted studies to substantiate the potential protective effects of androgen-deprivation therapy.

A more recent prospective longitudinal study of hospitalized males with COVID-19 suggested that longer AR CAG repeats are associated with a more severe form of the disease, supporting the active role of testosterone in the pathogenesis of the complicated disease (63).

Furthermore, there is evidence that AR has an impact on furin and other members of the convertase proprotein family in prostate cancer, which may support an alternative role for testosterone in the pathogenesis of COVID-19 (64, 65). This two-pronged position of testosterone to employ either TMPRSS2 or furin to intensify the virulence of SARS-CoV-2 warrants investigation in targeted studies.

Further to the AR genetic disparities among various ethnic populations, other natural candidate genetic polymorphisms related to *ACE2, TMPRSS2, or FURIN* genes, as well as other host invasion genes such as *DPP4 or PCSK3*, which have been shown to differ among different population ancestries, may also provide a supplementary explanation for COVID-19 pandemic spread and progression (66–68). It is possible that the presence of different *ACE2, TMPRSS2, FURIN*, *DPP4*, and *PCSK3* gene variants, the main machinery for orchestrating SARS-CoV-2 cellular host access, may modulate viral infectivity among humans, making some people less or more vulnerable than others.

Taken together, epidemiological data emerging from the COVID-19 pandemic, backed by animal studies and further by preliminary clinical studies in diverse clinical settings, support the notion that high (male) testosterone levels acting *via* the AR modulate TMPRSS2 function positively to further prime SARS-CoV-2 S proteins and eventually increase COVID-19 infectivity and severity. Additionally, as in various ethnic backgrounds, *AR* mutations or other gene polymorphisms along the pathway of SARS-Co-2 pathogenesis may further lead to COVID-19 expansion and deterioration. This concept ought to be further explored in properly performed targeted studies.

## Low Testosterone Impact on COVID-19 Severity—The ACE2 Connection

Serum testosterone levels decline with aging among men (69, 70) and the presence of comorbidities such as obesity, diabetes mellitus, cardiovascular disease, and chronic obstructive pulmonary disease, may further accentuate testosterone decrease among these men (71–74). Functional hypogonadism, which was previously referred to as “late-onset” hypogonadism, is a condition in which the endogenous secretion of testosterone is either insufficient or inadequate to maintain serum testosterone levels within the normal range, and may manifest as a variety of signs and symptoms. In addition to reduced sexual function, age-related hypogonadal men may have impaired energy, muscle mass and performance, cognitive function, bone mass with increased fracture risk, and anemia (74).

Age-related hypogonadism in men is due to a combination of primary hypogonadism (testicular insufficiency) and secondary hypogonadism (hypothalamic-pituitary insufficiency). While the first is the result of a reduced number of Leydig cells in the testis and less responsiveness of these cells to LH stimulation, the latter is the result of decreased GnRH production by the hypothalamus causing a decrease in LH secretion (75, 76). In these cases, serum T levels are low (below 10.5 nmol/L), however in some cases hypogonadism may be compensated, apparent by normal serum T level and high LH level.

Functional hypogonadism in adult men is often underdiagnosed and therefore undertreated. This has been explained as related symptoms are easily attributed to aging or other medical causes or ignored by patients and physicians. More than 60% of men over age 65 have free testosterone levels below the normal values of men aged 30 to 35. The community prevalence estimates of potentially functional hypogonadism in middle-aged and older men vary from 2.1% to 12.3%, with wide geographic and racial variation (74, 77). However, in men with comorbidities the prevalence may be much higher, reaching a rate of 22 to 69% in men with chronic obstructive pulmonary disease (71).

There are remarkable sex differences between the physiological mechanisms regulating arterial pressure, renal and vascular functions in humans (78–80). Accumulating evidence suggests that several components of the RAAS are regulated by sex hormones, as well as influenced by hormone replacement therapies (80). This is attributed to the differential balance in the pressor and the counterpart depressor arms of the RAAS in related organs. Mounting evidence suggests that sex hormones, androgens and estrogens, and modulation of the ACE2 expression may also take place in the lungs (81, 82). The *ACE2* gene is located on the X chromosome, with females generally having higher ACE2 activity than males (83), yet ACE2 expression levels in the lungs as well as in the myocardium have recently been demonstrated to be higher in males (53, 82). Furthermore, while *ACE2* gene expression decreases with age, it has been shown to have a negative correlation with COVID-19 severity and mortality (84). ACE2 is the receptor entry of SARS-CoV-2 infection and progression, but then again is a guardian against lung injury. It is, therefore, reasonable to speculate that in men with functional hypogonadism, low testosterone levels may aggravate COVID-19 infection and exacerbate morbidity and mortality.

RAAS components, and specifically ACE2, have also been shown to be involved in normal testosterone production, steroidogenesis, and spermatogenesis in mammalian animal models as well as in humans (85). Recently, ACE2 expression patterns were found to predominate spermatogonia, Leydig, and Sertoli cells in the adult human testis (86), offering evidence that the reproductive system in men is a potential target of COVID-19. There is some preliminary evidence to show that acute SARS-CoV-2 infection has the potential to infect the testes causing orchitis, and leading to a reduced ratio of serum testosterone to LH levels (87–89). This may potentially enhance the susceptibility of men with functional or borderline hypogonadism to COVID-19 infection and deterioration.

Moreover, testosterone is implicated in physiological processes in adult males other than reproduction and sexuality. Among these functions, testosterone has anti-inflammatory and immune-modulatory protective effects, achieved by regulating the differentiation of T lymphocytes (90–92). Androgens seem to be essential to mounting an anti-viral response and combating infection in males. Accumulating evidence suggests that in cases with severe SARS-CoV-2 infection, there is an acute disruption of the immune response. Specifically, secondary cytokine storm syndrome has been shown to complicate severe COVID-19 cases, leading to multiple-organ failure and mortality (93).

Furthermore, testosterone seems to have a modulatory influence on vascular integrity in aging men. Although most of the literature on sex differences has focused on the effects of estrogen deficiency associated with menopause and the protective effect of hormone replacement therapy, little attention has been paid to testosterone and its contribution to vascular aging. Accumulating data suggest that testosterone deficiency in aging men is related to endothelial dysfunction, arterial stiffness, and thrombocyte malfunction, predisposing men with COVID-19 to increased risk of venous and arterial thrombo-embolic phenomenon causing mortality (94, 95).

Indeed, two recent preliminary, unrelated cohort studies targeted patients with severe COVID-19, admitted to intensive care units, with a high rate of comorbidities. Both studies independently showed that low testosterone and dihydrotestosterone levels were correlated with COVID-19 severity and mortality (96, 97). Furthermore, low testosterone levels were found to correlate with high levels of inflammatory cytokines (96).

Taken together, low testosterone levels, a pathognomonic biomarker of aging males with functional hypogonadism, seems to be a substantial factor for poor prognosis and mortality in SARS-CoV-2 infected men. This may be substantially aggravated in men with co-morbidities admitted to intensive care units. Further studies are needed to substantiate this notion.

## Discussion

**Figure 1:**
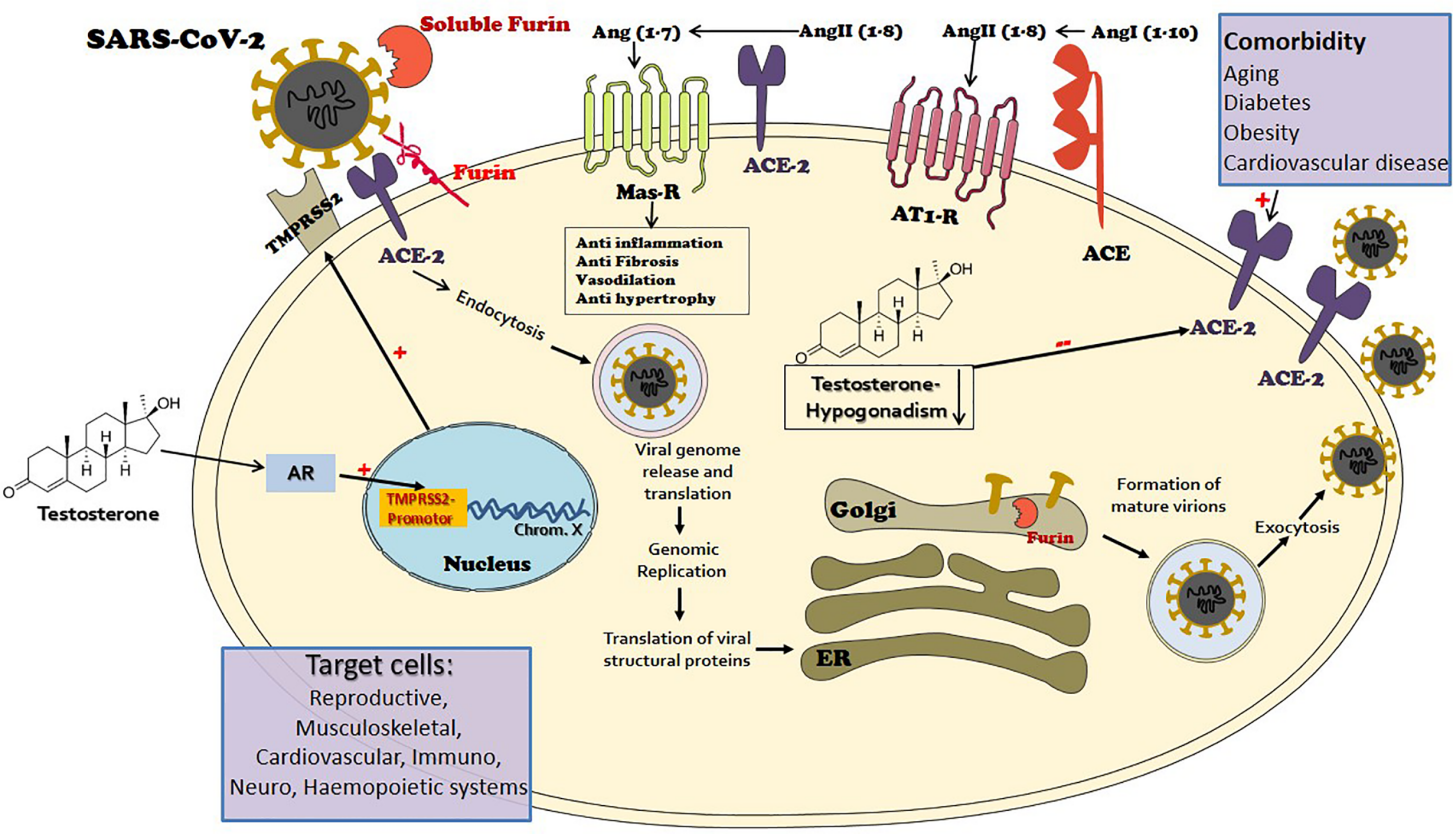
The port of entry of the novel mutant virus Severe Acute Respiratory Syndrome (SARS)-CoV-2 to target cells is *via* the angiotensin-converting enzyme 2 (ACE2), a key element of the renin-angiotensin-aldosterone system (RAAS). ACE2 is widely expressed in the human body and is largely responsible for the generation of angiotensin 1-7 from angiotensin II. The angiotensin 1-7-Mas receptor axis provokes beneficial balancing and salutary actions to counterpart the adverse branch of renin-angiotensin I-angiotensin II-AT1R axis in the RAAS, in vital organs such as the lung, heart, and kidney. The viral envelope bears transmembrane spike (S) glycoproteins applied to ACE2 attachment. Following ACE2 binding, cleavage of the viral spike protein (S) by proteases including transmembrane protease serine 2 (MPRSS2) and furin is considered as an essential step to effectuate host cell membrane fusion and virus infection. The priming process by TMPRSS2 seems to be vital for the entry of SARS-CoV-2 into human host cells. TMPRSS2 transcription is exclusively regulated by the androgen receptor (AR). The AR has a widespread expression in many tissues with a diverse range of biological actions, including the cardiovascular, reproductive, musculoskeletal, immune, neural, and haemopoietic systems. Male level testosterone seems to play a vital role in COVID-19 pathogenesis and severity, *via* the TMPRSS2 connection. While functional hypogonadism, a prevalent occurrence in aging men that is more widespread in men with comorbidities, also has an adversative role, *via* the ACE2 connection.

This mini review illustrates that COVID-19, an amply complex disease generated by the novel mutated SARS-CoV-2, has paved the way to explore numerous mechanisms of health and disease in the human body. Sex hormones, specifically testosterone seem to be a key factor in the development and spread of the disease. It seems that testosterone may be considered a double-edged sword in the pathogenesis of COVID-19 morbidity and mortality. In turn, the edges may correspond to countervailing connectors and pathways: TMPRSS2 promoting the contribution of testosterone excess and ACE-2 promoting the role of age-related testosterone deficiency (**Figure 1**). Epidemiological data, animal models and in-vitro cell experiments, and clinical studies support our conclusion. Unfolding the mechanisms and pathways of COVID-19 development and spread related to testosterone may open new horizons for disease containment, treatment, and eradication. This paper paves the way for several future directions of clinical, translational, and basic science investigations. **Table 1** summarizes the outstanding and unresolved questions that warrant examination in future targeted studies.

**Table 1:** Unresolved questions relating to the “double-edged” role of testosterone in COVID-19.

What is the relation between serum testosterone levels and biomarkers of severe COVID-19 such as: lymphocyte count, CRP, D-dimers, ferritin and IL-6 (98–100)?
What is the relation between testosterone levels, androgen receptor mutations/polymorphisms and TMPRSSE2 function in priming SARS-CoV-2 spike proteins, and in turn COVID-19 morbidity and mortality?
What is the relation between testosterone levels in men with age-related functional hypogonadism, COVID-19 and ACE2 expression, and in turn to disease severity and mortality, in men with co-morbidities or patients admitted to intensive care units?
Can precision guidance be used to consider whether testosterone replacement therapy or, conversely, testosterone deprivation drugs, in the appropriate settings, for management of patients with COVID-19?

## Author Contributions

JY and ZA created and developed the concept. JY drafted the manuscript. ZA and KS edited and revised the manuscript for important intellectual content. All authors contributed to the article and approved the submitted version.

## Conflict of Interest

The authors declare that the research was conducted in the absence of any commercial or financial relationships that could be construed as a potential conflict of interest.
